# First Zagreb energy of self-looped graphs: predictive insights into kidney infection drugs and theoretical bounds

**DOI:** 10.1038/s41598-026-45777-7

**Published:** 2026-04-08

**Authors:** L. Yashaswini, B. R. Rakshith, P. Nagaraja

**Affiliations:** Manipal Institute of Technology, Manipal Academy of Higher Education, Manipal, India

**Keywords:** First Zagreb matrix, Energy, Self-loop graphs, Chemistry, Mathematics and computing

## Abstract

Graphs containing self-loops provide a versatile framework for modeling heteroatomic molecules, with each self-loop representing a hetero-atom. In this study, we investigate the predictive capability of the first Zagreb energy in relation to the physicochemical properties of kidney infection drugs, using their corresponding molecular graphs with self-loops. The analysis using linear, quadratic, cubic, and logarithmic regression models reveals a strong correlation between the first Zagreb energy and key physicochemical properties such as polarizability, molar refractivity, and molar volume. Statistical metrics such as standard error (SE), F-test value, standard error of fit (SF), and root mean squared error (RMSE) validate the stability and reliability of the proposed models. Furthermore, we compute the first Zagreb energy of the complete graph $(K_{n})_{_S}$, as well as the complete bipartite graph $(K_{m,n})_{_S}$, with partite sets $M=S$, and *N*. In addition, we derive both lower and upper bounds for the first Zagreb energy of graphs with self-loops.

## Introduction

Let *G* be a simple, undirected graph with vertex set *V*(*G*), where $|V(G)|=n$, and edge set *E*(*G*) with $|E(G)|=m$. The vertices of *G* are labeled as $v_{1},v_{2},\ldots ,v_{n}$. We write $v_{i}\sim v_{j}$, or simply $i\sim j$ if $v_{i}$ is adjacent to $v_{j}$. For a subset *S* of *V*(*G*), the self-loop graph $G_{S}$ is obtained from *G* by adding a self-loop to each vertex of *G* which are in the set *S*. We denote the cardinality of the set *S* by $\sigma$. A graph *G* with $\sigma$ self-loops is denoted by $G^{\sigma }$. The degree of a vertex $v_{i}$ in *G* is denoted by $d_{i}$, and its degree in $G_{S}$ is denoted as $d^{\prime }_{i}$. We have $d^{\prime }_{i}=d_{i}$ if $v_{i}\notin S$, and $d^{\prime }_{i}=d_{i}+2$ if $v_{i}\in S$. Recently, Gutman et al.^1^ introduced the adjacency matrix for graphs with self-loops, denoted as $A(G_{S})=(a_{i})_{n\times n}$, where $a_{ij}=1$ if $v_{i}\sim v_{j}$, or if $i=j$ and $v_{i}\in S$, and 0 otherwise. The eigenvalues of $A(G_{S})$ are referred as eigenvalues of graph $G_{S}$. Let $\lambda ^{S}_{i}$ denote the *i*-th largest eigenvalue of $G_{S}$. The energy of $G_{S}$, denoted by $\mathcal {E}(G_{S})$, refers to the quantity $\sum _{i=1}^{n}|\lambda _{i}^{S}-\dfrac{\sigma }{n}|$. This definition was proposed in conjunction with the adjacency matrix of self-loop graphs in^1^. For more details, see^1–4^.

Topological indices are the numerical values derived from the graph structures. It quantifies the structural connectivity of the graph and acts as a mathematical tool to represent and analyze molecular properties. For more information, see^5–12^. Degree based topological indices are of the form $TI=\sum _{v_{i}\sim v_{j}}f(d_{i},d_{j})$, where *f* is a non-negative real symmetric function of two variables^13,14^. A topological matrix is a graph matrix derived from a topological index. For the index *TI* as defined above, its topological matrix is given by $TI(G)=(f_{ij})_{n\times n}$, where $f_{ij}=f(d_{i},d_{j})$ if $v_{i}\sim v_{j}$, and 0 otherwise^13^. Some of the well-known topological matrices are atom-bond connectivity matrix, first Zagreb matrix, geometric-arithmetic matrix, Randić matrix, sum connectivity matrix, Sombor matrix, etc. For details, see^13–19^. Recently, the concept of energy of graphs with self-loops is extended to various topological matrices. The degree based topological matrix of a self loop graph $G_{S}$ is given by $TI(G_{S})=(f_{ij})$, where $f_{ij}=f(d^{\prime }_{i},d^{\prime }_{j})$ if $v_{i}\sim v_{j}$, or if $i=j$ and $v_{i}\in S$, and 0 otherwise. The energy of $G_{S}$ associated with the topological matrix $TI(G_{S})$ is the sum $\sum _{i=1}^{n}|\theta _{i}-\dfrac{trace(TI)}{n}|$, where $\theta _{i}$ for $i=1,2,3\ldots ,n$ are the eigenvalues of $TI(G_{S})$, see^20,21^. Deekshitha et al. in^22^ introduced Sombor energy of a self-loop graph and studied its mathematical properties, and also explored its correlation with total $\pi$-electron energies associated with the corresponding hetero-molecular systems. In^23^, Sharath et al. considered atom bond connectivity energy of self-loop graphs and explored its application to structure property relationships in anticancer drugs.

One of the classical topological indices is the first Zagreb index introduced in^24^. It is one of the most extensively studied topological indices, denoted by $M_{1}(G)$ and defined as

$$\begin{aligned} M_{1}(G)=\sum \limits _{v_i\sim v_j}{d_i+d_j}=\sum \limits _{v_i\in V(G)}d_i^2. \end{aligned}$$

The first Zagreb matrix of *G* is a topological matrix derived from the first Zagreb index $M_{1}(G)$. It is denoted by $Z_{1}(G)$, and is defined as $Z_{1}(G) =(z_{ij})$, where $z_{ij}= d_i+d_j$ if $v_i \sim v_j$, and 0 if $v_i \not \sim v_j$^25^. The first Zagreb energy of *G*, $\mathcal {E}_{Z_{1}}(G)$ is defined as the sum of absolute values of all the eigenvalues of $Z_{1}(G)$. Studies on first Zagreb matrix and its energy can be found in^25,26^. The first Zagreb index is one of the oldest degree-based topological indices, with significant applications in chemistry. The first Zagreb matrix has also received extensive study. It is therefore logical to explore the first Zagreb matrix of graphs with self-loops to investigate its structural and spectral properties, following recent work on the Sombor matrix with self-loops and the Atom–Bond Connectivity matrix with self-loops. The first Zagreb matrix of the self-loop graph $G_{S}$ is defined as $Z_{1}(G_{S})=(z_{ij})_{n\times n}$, where

$$\begin{aligned} z_{ij}={\left\{ \begin{array}{ll}d^{\prime }_{i}+d^{\prime }_{j}, & \text {if}\, v_{i}\sim v_{j},\, \text {or}\, i=j\, \text {and}\, v_{i}\in S\\ 0, & otherwise.\end{array}\right. } \end{aligned}$$

The eigenvalues of $Z_{1}(G_{S})$ are denoted by $\zeta _{i}$ for $i=1,2,\ldots ,n$, with $\zeta _{i}$ being the *i*-th largest eigenvalue of $Z_{1}(G_{S})$. In conjunction with the first Zagreb matrix $Z_{1}(G_{S})$, we define the first Zagreb energy of $G_{S}$ as $\mathcal {E}(G_{S})=\sum _{i=1}^{n}\Big | \zeta _i- \dfrac{2\mathcal {D_{S}}}{n}\Big |,$ where $\mathcal {D_S}=\sum \limits _{v_i\in S}d^{\prime }_i$. In “QSPR analysis of kidney infection drugs”, of the paper, we explore the chemical significance of first Zagreb energy of molecular graphs with self-loops by performing QSPR analysis of kidney infection drugs in comparison with first Zagreb energy of molecular graphs with no self-loops . The analysis using linear, quadratic, cubic, and logarithmic regression models reveals a strong correlation between the first Zagreb energy and key physicochemical properties such as polarizability, molar refractivity, and molar volume. In Sect. 3, we compute the first Zagreb energy of the complete graph $(K_{n})_{S}$, as well as the complete bipartite graph $(K_{m,n})_{S}$, with partite sets *M* and *N*, with $M=S$. In Sect. 4, we derive bounds for the first Zagreb energy $\mathcal {E}(G_S)$.

Section “QSPR analysis of kidney infection drugs” establishes the applicability of the first Zagreb energy of self-looped graphs as a molecular descriptor through its analysis of kidney infection drugs. The theoretical bounds obtained in the study provide insight into the possible range of the descriptor values. Thus, the applied perspective presented in “QSPR analysis of kidney infection drugs” is supported and strengthened by the theoretical results in the later sections, ensuring both mathematical rigor and chemical relevance. This study demonstrates the chemical significance of the first Zagreb energy by incorporating self-loops into molecular graphs. This extension of traditional chemical graph models effectively represents heteroatomic molecules. It is shown that, in the case of kidney infection drugs, the predictive ability of the first Zagreb energy of molecular graphs with self-loops is better than that of the first Zagreb energy of molecular graphs without self-loops.

## QSPR analysis of kidney infection drugs

Hückel molecular orbital theory has been predominantly developed with a focus on conjugated compounds composed entirely of carbon atoms. The scope of such compounds can be broadened by incorporating hetero-atoms into the analysis. Chemists have extensively studied various topological indices to establish correlations between the structure of chemical compounds and their experimentally determined physicochemical properties. Graphs that include self-loops provide a versatile framework for studying hetero-atomic molecules, with each self-loop representing a hetero atom. QSPR analysis helps in discovery and development of drug by linking chemical structures with their physicochemical properties. It predicts the molecular behaviors and makes drug design faster and more efficient.

**Fig. 1:**
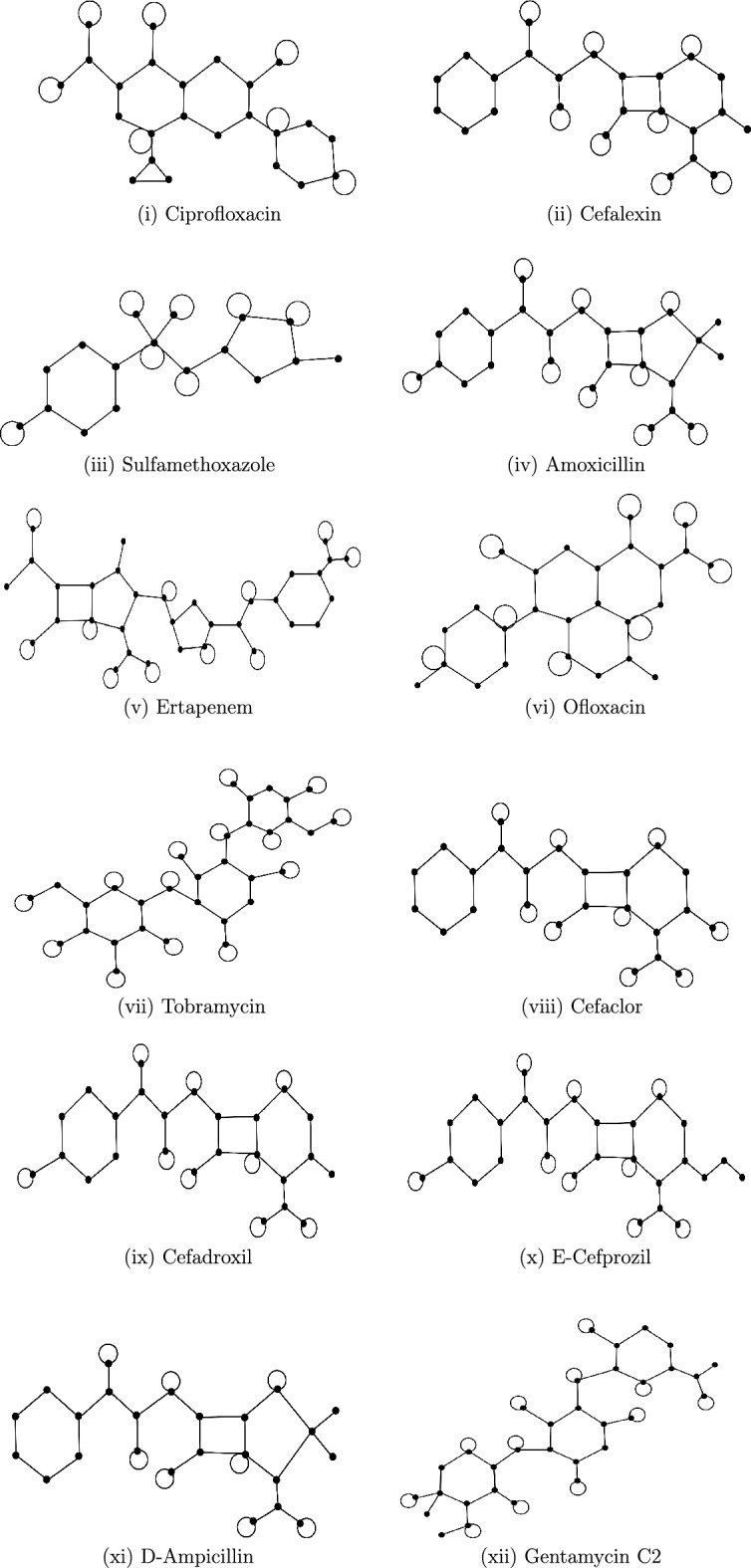
Molecular structures of drugs used in the treatment of kidney infection.

In this section, we consider the drugs, namely Ciprofloxacin, Cefalexin, Sulfamethoxazole, Amoxicillin, Ertapenem, Ofloxacin, Tobramycin, Cefaclor, Cefadroxil, E-Cefprozil, D-ampicillin and Gentamicin C2, which are used as medications for kidney infections. The molecular graphs of these drugs, available in ChemSpider repository, are shown in Fig. 1 and are also reported in^7^. The chemical compounds the corresponding values of their physicochemical properties were obtained from the same repository and are listed in Table 1 (see also^7^). We use vertex and edge partition technique, and the MATLAB software to determine the first Zagreb energy of molecular graphs with and without self-loops. The computed energies are given in Table 2. The physicochemical characteristics of drugs such as boiling point ($BP$), flashpoint ($FP$), enthalpy of vaporization ($E$), molar refractivity ($MR$), molar volume ($MV$), polarizability ($P$), and polar surface area ($PSA$) are considered for the analysis. Various regression analysis are carried out through Microsoft Excel to assess the effectiveness of first Zagreb energy as structural descriptors. The following regression equations are considered for the study.

2.1 $$\begin{aligned} Y&= a X + b. \quad \textit{(Linear equation)} \nonumber \\ Y&= a X^2 + b X+c. \quad \textit{(Quadratic equation)} \nonumber \\ Y&= a X^3 + b X^2+c X+d. \quad \textit{(Cubic equation)} \nonumber \\ Y&=a\ln X+b. \quad \textit{(Logarithmic equation)} \end{aligned}$$

**Table 1:** Physicochemical properties of drugs used in the treatment of kidney infections.

Drugs	BP (${}^\circ$C)	E (kJ/mol)	FP (${}^\circ$C)	MR ($\textrm{cm}^3$)	PSA (Å$^2$)	P($10^{-24}$ $\text {cm}^3$)	MV ($\text {cm}^3$)
Ciprofloxacin	581.8	91.5	305.6	83.3	73	33.0	226.8
Cefalexin	727.4	11.5	393.7	89.4	138	34.5	231.3
Sulfamethoxazole	482.1	74.7	245.4	62.5	107	24.8	173.1
Amoxicillin	743.2	113.7	403.1	91.5	158	36.8	236.2
Ertapenem	813.9	124.0	446.0	118.3	182	46.9	306.2
Ofloxacin	571.5	90.1	299.4	91.1	73	36.1	244.0
Tobramycin	775.4	128.7	422.8	111.7	268	44.4	305.9
Cefaclor	713.4	109.5	385.2	89.2	133	35.5	225.6
Cefadroxil	789.9	120.5	431.5	90.9	158	36.1	232.6
E-Cefprozil	803.1	122.4	439.5	100.1	158	39.7	253.9
D-ampicillin	683.9	105.4	367.4	89.9	138	35.7	239.3
Gentamicin C2	676.3	113.6	362.8	118.0	214	46.8	346.8

**Table 2:** First Zagreb energy of molecular graphs and first Zagreb energy of molecular graphs with self-loops.

Drugs	$\mathcal {E}_{ Z_1}(G_S)$	$\mathcal {E}_{ Z_1}(G)$
Ciprofloxacin	200.5	153.32
Cefalexin	187.9	145.25
Sulfamethoxazole	138.93	99.0
Amoxicillin	203.89	151.6
Ertapenem	255.23	193.54
Ofloxacin	218.67	164.68
Tobramycin	257.9	188.7
Cefaclor	193.57	145.25
Cefadroxil	196.9	150.4
E-Cefprozil	212.4	160.6
D-ampicillin	194.48	146.42
Gentamicin C2	256.0	189.19

**Regression models:**

Table 3 lists the linear regression equations of *BP*, *E*, *FP*, *MR*, *PSA*, *P*, and *MV* in terms of first Zagreb energy, with and without self-loops, along with the associated standard error (*SE*), *F*-test value (*F*), significance level (*SF*), correlation coefficient (*R*), and the root mean squared error (*RMSE*).

**Table 3:** Linear regression equations and statistical parameters.

Linear regression equation	R	SE	F	SF	RMSE
$BP = 1.3\,\mathcal {E}_{ Z_1}(G_S)-21.639$	0.5476	91.2	4.284	0.065	14.1073
$BP = 2.3415\,\mathcal {E}_{ Z_1}(G)+328.24$	0.5824	88.605	5.135	0.046	80.88
$E = 0.5\,\mathcal {E}_{ Z_1}(G_S)-2.5$	0.5197	28.9	3.7	0.08	83.2624
$E = 0.601\,\mathcal {E}_{ Z_1}(G)+5.86$	0.4815	29.66	3.019	0.1129	27.07
$FP = 1.0075\,\mathcal {E}_{ Z_1}(G_S)+163.87$	0.5476	55.16	4.28	0.65	50.359
$FP = 1.416\,\mathcal {E}_{ Z_1}(G)+152.32$	0.5824	53.59	5.133	0.047	48.92
$MR = 0.4415\,\mathcal {E}_{ Z_1}(G_S)+2.0758$	**0.9629**	**4.43**	**127.36**	$5.19\times 10^{-7}$	**4.047**
$MR=0.5829\,\mathcal {E}_{ Z_1}(G)+2.93$	0.9621	4.4785	124.6	$5.8\times 10^{-7}$	4.0883
$PSA = 1.063\,\mathcal {E}_{ Z_1}(G_S)-72.58$	0.6594	43.43	7.7	0.0196	39.6463
$PSA = 1.309\,\mathcal {E}_{ Z_1}(G)-55.74$	0.6148	45.56	6.076	0.033	41.59
$P = 0.1765\,\mathcal {E}_{ Z_1}(G_S)+0.4417$	**0.9658**	**1.7**	**138.69**	$3.48\times 10^{-7}$	**1.55**
$P=0.2325\,\mathcal {E}_{ Z_1}(G)+0.8738$	0.9626	1.774	126.337	$5.4\times 10^{-7}$	1.62
$MV = 1.3\,\mathcal {E}_{ Z_1}(G_S)-21.639$	**0.9492**	**15.45**	**90.87**	$2.46\times 10^{-6}$	**14.1073**
$MV=1.6827\,\mathcal {E}_{ Z_1}(G)-13.814$	0.9297	18.07	63.748	$1.2\times 10^{-5}$	16.4986

**Table 4:** The correlation coefficients of $\mathcal {E}_{ Z_1}(G_S)$ and $\mathcal {E}_{ Z_1}(G)$ with the physicochemical characteristics computed by linear regression equations.

*R*	BP	E	FP	MR (cm$^3$)	PSA (Å$^2$)	P(10$^{-24}$ cm$^3$)	MV (cm$^3$)
$\mathcal {E}_{ Z_1}(G_S)$	0.5476	0.5197	0.5476	**0.9629**	0.6594	**0.9658**	**0.9492**
$\mathcal {E}_{ Z_1}(G)$	0.5825	0.4185	0.5824	0.9621	0.6148	0.9626	0.9297

From the data listed in Table 4, one can observe that first Zagreb energy of molecular graphs with self-loops shows higher correlation with polarizability ($P$), molar refractivity ($MR$) and molar volume ($MV$). Experimental values of *MR*, *P*, and *MV*, along with their corresponding values predicted by the linear regression models, are presented in Table 5. The results indicate a strong correlation between the experimental and predicted values of *MR*, *P*, and *MV*, demonstrating the good predictive capability of the proposed models.

**Table 5:** Comparison of experimental and computed values from linear regression models.

Drugs	MR(cm$^{3}$)	Predicted MR	P($10^{-24}$ cm$^{3}$)	Predicted P	MV(cm$^{3}$)	Predicted MV
Ciprofloxacin	83.3	90.59	33.0	35.8386	226.8	244.1774
Cefalexin	89.4	85.036	34.5	33.6142	231.3	230.5981
Sulfamethoxazole	62.5	63.415	24.8	24.9689	173.1	152.7739
Amoxicillin	91.5	92.096	36.3	36.4371	236.2	241.2832
Ertapenem	118.3	114.763	46.9	45.5008	306.2	311.8555
Ofloxacin	91.1	98.6215	36.1	39.0464	244.0	263.1582
Tobramycin	111.7	115.8025	44.3	45.9474	305.9	305.5622
Cefaclor	89.6	87.9810	35.5	34.7917	226.5	230.5981
Cefadroxil	89.0	89.0098	35.5	35.2030	222.8	239.2639
E-cefprozil	100.1	95.8532	38.7	37.9395	253.9	256.4275
D-ampicillin	89.9	87.9413	35.7	34.7758	239.3	232.5668
Gentamicin C2	118.0	115.1032	46.8	45.6367	346.8	304.5357

**Fig. 2:**
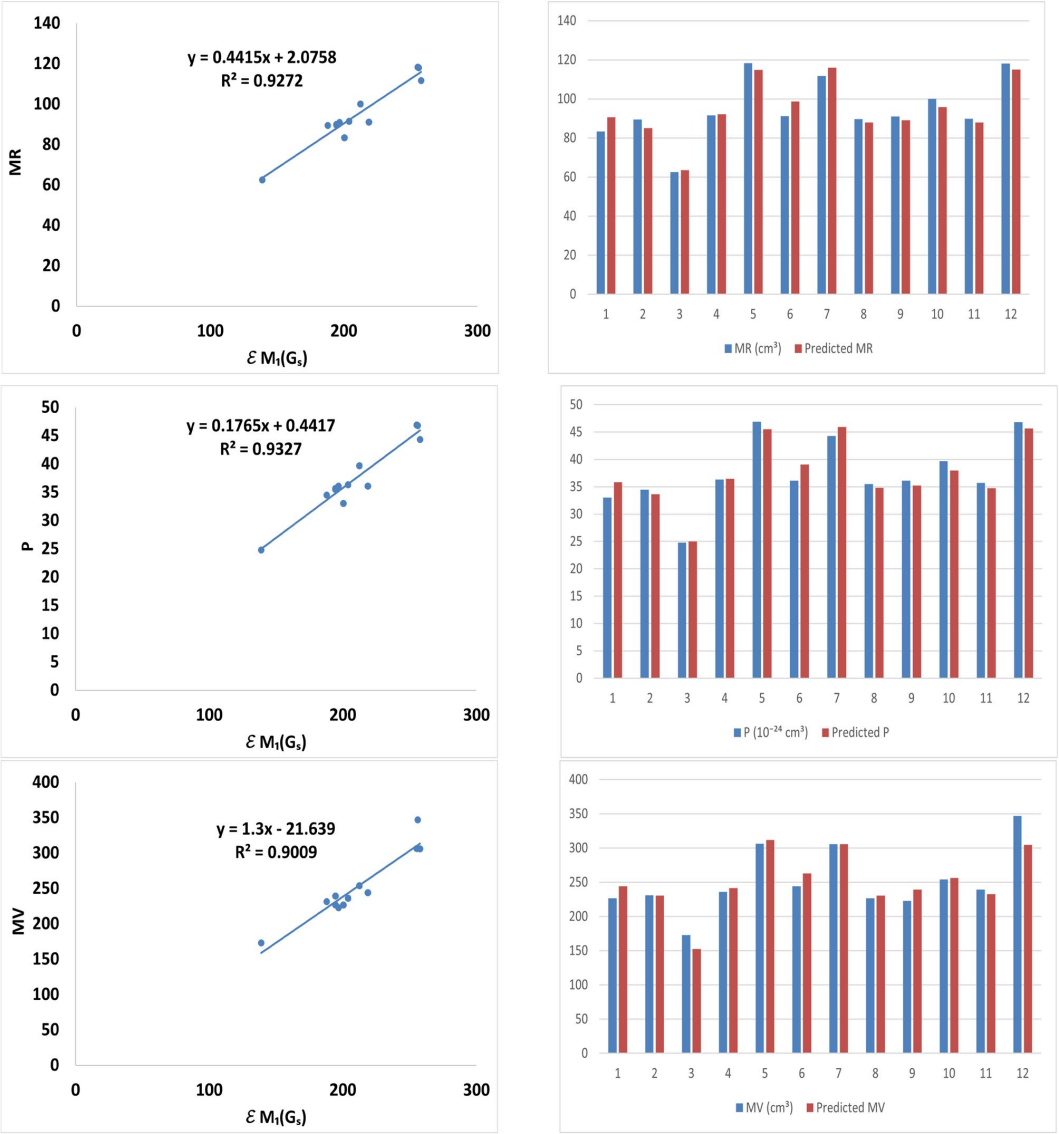
Linear regression of $\mathcal {E}_{ Z_1}(G_S)$ with $MR$, $P$ and $MV$ and, observed and predicted values of respective physicochemical properties of drugs.

Figure 2 illustrates the linear regression relationships between $\mathcal {E}{Z_1}(G_S)$ and *MR*, *P*, and *MV*, respectively. A strong correlation between the experimental and predicted values of *MR*, *P*, and *MV* with respect to $\mathcal {E}{Z_1}(G_S)$ is also presented in Fig. 2 using bar graphs. In these graphs, the experimental values are represented in blue, while the predicted values are shown in red.

**Table 6:** Quadratic regression equations and statistical parameters.

Quadratic regression equation	R	SE	F	SF	RMSE
$MR=5 \times 10^{-5} \,\mathcal {E}^{2}_{ Z_1}(G_S)+0.4205 \,\mathcal {E}_{ Z_1}(G_S)+4.1963$	0.9629	4.67	57.334	$7.57 \times 10^{-6}$	4.046
$MR=0.0013 \,\mathcal {E}^{2}_{ Z_1}(G)+0.1914\, \mathcal {E}_{ Z_1}(G)+31.686$	0.9653	4.52	61.52	$5.63 \times 10^{-6}$	3.9157
$P=5 \times 10^{-5} \,\mathcal {E}^{2}_{ Z_1}(G_S)+0.1568\, \mathcal {E}_{ Z_1}(G_S)+2.4322$	0.9658	1.788	62.54	$5.26 \times 10^{-6}$	1.55
$P=0.0006 \,\mathcal {E}^{2}_{ Z_1}(G)+0.0593\, \mathcal {E}_{ Z_1}(G)+13.6$	0.9665	1.77	63.9	$4.8 \times 10^{-6}$	1.5337
$MV=0.0048 \,\mathcal {E}^{2}_{ Z_1}(G_S)-0.6705\, \mathcal {E}_{ Z_1}(G_S)+177.14$	**0.9617**	14.17	55.42	$8.71 \times 10^{-6}$	43.01
$MV=0.0104 \,\mathcal {E}^{2}_{ Z_1}(G)+1.4673\, \mathcal {E}_{ Z_1}(G)+217.56$	0.9534	15.6	44.92	$2.07 \times 10^{-5}$	13.52

In the following we consider quadratic, cubic, and logarithmic regression models for *MR*, *P*, and *MV*. In quadratic regression analysis, $\mathcal {E}_{ Z_1}(G_S)$ exhibits strong correlation with $MV$ when compared to $\mathcal {E}_{ Z_1}(G)$.

**Table 7:** Cubic regression equations and statistical parameters.

Cubic regression equation	R	SE	F	SF	RMSE
$MR=7 \times 10^{-5}\,\mathcal {E}^{3}_{ Z_1}(G_S)-0.0388\,\mathcal {E}^{2}_{ Z_1}(G_S)+7.93\,\mathcal {E}_{ Z_1}(G_S)-465.22$	0.9687	4.56	40.6	$3.46 \times 10^{-5}$	3.724
$MR=0.0002\,\mathcal {E}^{3}_{ Z_1}(G)-0.0685\,\mathcal {E}^{2}_{ Z_1}(G)+10.143\,\mathcal {E}_{ Z_1}(G)-423.6$	0.9719	4.32	45.57	$2.2 \times 10^{-5}$	3.526
$P=2 \times 10^{-5}\,\mathcal {E}^{3}_{ Z_1}(G_S)-0.0123\,\mathcal {E}^{2}_{ Z_1}(G_S)+2.5473\,\mathcal {E}_{ Z_1}(G_S)-147.02$	0.97	1.79	41.76	$3.11 \times 10^{-5}$	1.465
$P=5 \times 10^{-5}\,\mathcal {E}^{3}_{ Z_1}(G)-0.0235\,\mathcal {E}^{2}_{ Z_1}(G)+3.486\,\mathcal {E}_{ Z_1}(G)-143.19$	0.9715	1.736	44.8	$2.4 \times 10^{-5}$	1.417
$MV=0.0002\,\mathcal {E}^{3}_{ Z_1}(G_S)-0.0931\,\mathcal {E}^{2}_{ Z_1}(G_S)+18.263\,\mathcal {E}_{ Z_1}(G_S)-1006.6$	**0.966**	14.22	37.04	$4.9 \times 10^{-5}$	11.61
$MV=0.0001\,\mathcal {E}^{3}_{ Z_1}(G)-0.0528\,\mathcal {E}^{2}_{ Z_1}(G)+7.5468\,\mathcal {E}_{ Z_1}(G)-194.83$	0.954	16.45	27	0.00015	13.43

Cubic regression analysis shows a stronger correlation between $\mathcal {E}_{Z_1}(G_S)$ and *MV* than between $\mathcal {E}_{Z_1}(G)$ and *MV*.

**Table 8:** Logarithmic regression equations and statistical parameters.

Logarithmic regression equation	R	SE	F	SF	RMSE
$MR=87.747\,\ln (\mathcal {E}_{ Z_1}(G_S))-373.27$	**0.9562**	4.8	106.881	$1.2 \times 10^{-6}$	4.3872
$MR=83.069\,\ln (\mathcal {E}_{ Z_1}(G))-324.41$	0.9446	5.39	82.844	$3.7 \times 10^{-6}$	4.922
$P=35.052\,\ln (\mathcal {E}_{ Z_1}(G_S))-149.46$	**0.9581**	1.87	112.123	$9.4 \times 10^{-7}$	1.71108
$P=33.091\,\ln (\mathcal {E}_{ Z_1}(G))-129.48$	0.9438	2.163	81.66	$4 \times 10^{-6}$	1.975
$MV=253.47\,\ln (\mathcal {E}_{ Z_1}(G_S))-1100.7$	**0.9247**	18.68	59.024	$1.7 \times 10^{-5}$	17.0539
$MV=235.06\,\ln (\mathcal {E}_{ Z_1}(G))-934.87$	0.8948	21.9	40.17	$8.5 \times 10^{-5}$	20

In logarithmic regression analysis, we observe strong correlation between $\mathcal {E}_{ Z_1}(G_S)$ and $MV$, $P$ and $MR$, with correlation coefficients **0.9562**, **0.9581**, and **0.9247**, respectively.

**Fig. 3:**
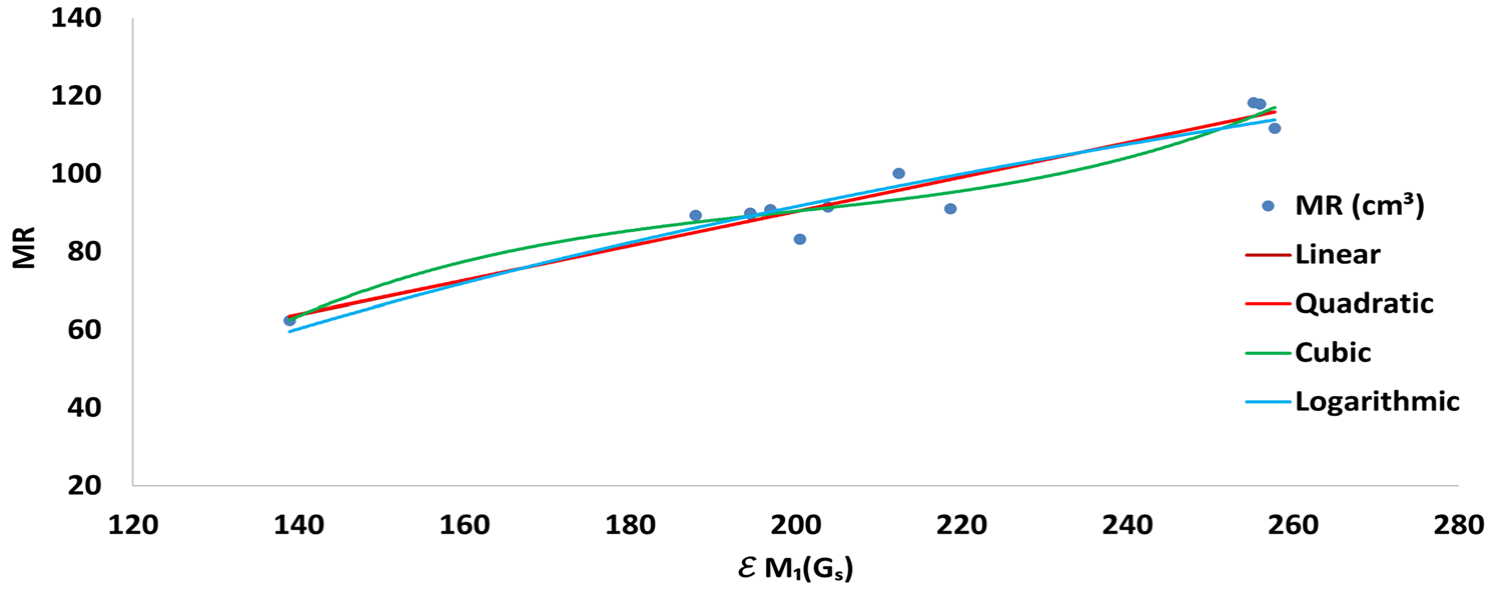
Correlation of $\mathcal {E}_{ Z_1}(G_S)$ with $MR$.

**Fig. 4:**
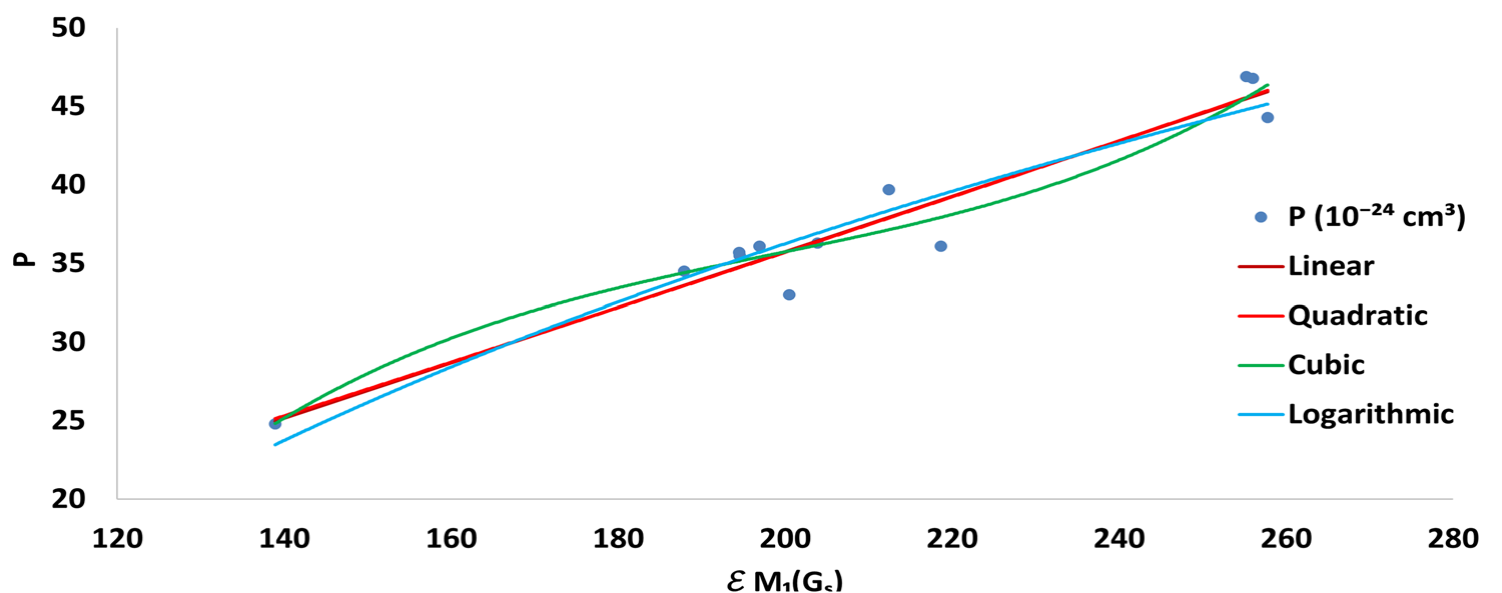
Correlation of $\mathcal {E}_{ Z_1}(G_S)$ with $P$.

**Fig. 5:**
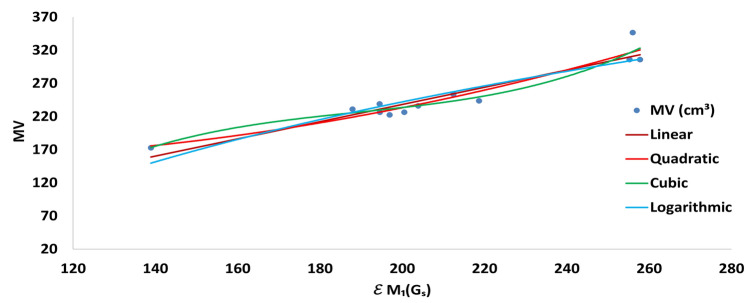
Correlation of $\mathcal {E}_{ Z_1}(G_S)$ with $MV$.

In most of these models (see Tables 3, 6, 7, and 8), the first Zagreb energy $\mathcal {E}_{Z_1}(G_S)$ exhibits a strong correlation with various physicochemical properties of drugs used in the treatment of kidney infections, compared to the corresponding energy $\mathcal {E}_{Z_1}(G)$. Figures 3, 4, and 5 illustrate the curvilinear variations in the linear, quadratic, cubic, and logarithmic fittings between $\mathcal {E}_{Z_1}(G_S)$ and the molecular properties *MR*, *P*, and *MV*, respectively. The correlations between $\mathcal {E}_{Z_1}(G_S)$ and *MR*, *P*, and *MV* are statistically reliable, showing strong correlations, low errors, highly significant *p*-values, and low *RMSE* values. Lower standard error and *RMSE* values indicate a stronger regression relationship, and when the significance factor (*SF*) is less than 0.05 while the correlation coefficient (*R*) exceeds the critical value of 0.5324, the model can be considered statistically reliable.

## First Zagreb energy of some graphs with $\sigma$-self loops

In this section, we determine the spectrum of the complete graph $K_n^{\sigma }$ and the complete bipartite graph $K_{m,n}^{\sigma }$, both with $\sigma$-self loops. The following lemma is important to prove our results. The all-ones matrix of order *n* is denoted by $J_{n}$, and that of order $n\times m$ is denoted by $J_{n\times m}$.

### Lemma 3.1

^27^ Let *N* be a block matrix of the form

$$N=\begin{bmatrix} N_1 & N_2 \\ N_3 & N_4 \\ \end{bmatrix}$$

where $N_1$ and $N_4$ are square matrices. If $N_1$ is invertible, i.e., ${det(N_{1})\ne 0}$, then

$$\det N = \det N_1 \cdot \det (N_4 - N_3 N_1^{-1} N_2).$$

Further, if $N_4$ is invertible, i.e., ${det(N_{4})\ne 0}$, then

$$\det N = \det N_4 \cdot \det (N_1 - N_3 N_4^{-1} N_2).$$

### Theorem 3.1

The first Zagreb energy of a complete graph $K^\sigma _n$ with $\sigma$-self loops is given by

$$\begin{aligned} \mathcal {E}_{ Z_1}(K_n^\sigma )=2(n^2-2n+1)- \dfrac{4\sigma }{n}+2\sqrt{4n(2n-1)\sigma +(n-1)^4} \end{aligned}$$

for $$1 \le \sigma \le n-1$$.

### Proof

Without loss of generality, we can assume that $S=\{v_{1},v_{2},\ldots ,v_{\sigma }\}$. Then the degree of $v_{i}$ is $n+1$ for $1\le i\le \sigma$, and the degree of $v_{i}$ for $i=\sigma +1, \sigma +2,\ldots ,n$ is $n-1$. Thus the (*i*, *j*)-th entry in $Z_{1}(K^\sigma _{n})$ is as follows.

$$(Z_{1}(K_{n}^{\sigma }))_{ij}=\left\{ \begin{array}{cc} 2(n+1), & 1\le i\le j\le \sigma \\ 2(n-1), & \sigma< i< j\le n\\ 2n, & 1\le i\le \sigma \,\, \text {and}\,\, \sigma< j\le n \\ 0, & i=j\,\, \text {and}\,\, \sigma < i\le n \end{array}\right. .$$

Hence,

$$Z_1(K^\sigma _n)=\begin{bmatrix} (2n+2)\,J_{\sigma } & 2n\,J_{\sigma \times (n-\sigma )}\\ 2n\,J_{(n-\sigma ) \times \sigma } & 2(n-1)\,(J-I)_{n-\sigma } \end{bmatrix}.$$

So,

$$| \zeta \,I-Z_1(K^\sigma _n)|=\begin{bmatrix} \zeta \,I_{\sigma }-(2n+2)\,J_{\sigma } & -2n\,J_{\sigma \times (n-\sigma )}\\ -2n\,J_{(n-\sigma ) \times \sigma } & \zeta \,I_{n-\sigma }-2(n-1)\,(J-I)_{n-\sigma } \end{bmatrix}.$$

By Lemma 3.1,

3.1 $$\begin{aligned} | \zeta \,I-Z_1(K^\sigma _n)|&=| \zeta \,I_{\sigma }-(2n+2)\,J_{\sigma }|\times \nonumber \\&| \zeta \,I_{n-\sigma }-2(n-1)\,(J-I)_{n-\sigma }-2n\,J_{(n-\sigma ) \times \sigma }( \zeta \,I_{\sigma }-(2n+2)\,J_{\sigma })^{-1}2n\,J_{\sigma \times (n-\sigma )}|. \end{aligned}$$

We have,

$$\begin{aligned} ( \zeta \,I_{\sigma }-(2n+2)\,J_{\sigma })J_{\sigma \times (n-\sigma )}=( \zeta -(2n+2)\sigma )J_{\sigma \times (n-\sigma )}. \end{aligned}$$

Therefore,

$$\begin{aligned} J_{\sigma \times (n-\sigma )}=( \zeta -(2n+2)\sigma )( \zeta \,I_{\sigma }-(2n+2)\,J_{\sigma })^{-1}J_{\sigma \times (n-\sigma )}, \end{aligned}$$

and so

3.2 $$\begin{aligned} J_{(n-\sigma )\times \sigma }( \zeta \,I_{\sigma }-(2n+2)\,J_{\sigma })^{-1}J_{\sigma \times (n-\sigma )}=\dfrac{\sigma \,J_{n-\sigma }}{ \zeta -(2n+2)\sigma }. \end{aligned}$$

Employing (3.2) in (3.1), we have

$$\begin{aligned} | \zeta \,I-Z_1(K^\sigma _n)|&=| \zeta \,I_{\sigma }-(2n+2)\,J_{\sigma }|\Big | \zeta \,I_{n-\sigma }-2(n-1)\,(J-I)_{n-\sigma }-\dfrac{4n^2\sigma \,J_{n-\sigma }}{ \zeta -(2n+2)\sigma }\Big |\\&= \zeta ^{\sigma -1}( \zeta +2(n-1))^{n-\sigma -1}( \zeta ^2-2(n^2-2n+2\sigma +1) \zeta -4\sigma (n^2+n-\sigma -1). \end{aligned}$$

Hence, the spectrum of $Z_1(K_n^\sigma )$ is

$$Spec[Z_1({K^\sigma _n})]=\begin{pmatrix} 0 & -2(n-1) & n^2-2n+2\sigma +1\pm \sqrt{4n(2n-1)\sigma +(n-1)^4} & \\ \sigma -1 & n-\sigma -1 & 1 \end{pmatrix}$$.

Now,

$$\begin{aligned} \mathcal {E}_{ Z_1}(K_n^\sigma )&=\sum \limits _{i=1}^{n}\Big | \zeta _i(K_{n}^\sigma )- \dfrac{2\sigma (n+1)}{n}\Big |\\&=(\sigma -1) \dfrac{2\sigma (n+1)}{n}+(n-\sigma -1)\Big (2n-2+ \dfrac{2\sigma (n+1)}{n}\Big )\\ &+\Big |n^2-2n+2\sigma +1\pm \sqrt{4n(2n-1)\sigma +(n-1)^4}- \dfrac{2\sigma (n+1)}{n}\Big | . \end{aligned}$$

Upon simplification,

$$\begin{aligned} \mathcal {E}_{ Z_1}(K_n^\sigma )=2(n^2-2n+1)- \dfrac{4\sigma }{n}+2\sqrt{4n(2n-1)\sigma +(n-1)^4}. \end{aligned}$$

$$\square$$

### Proposition 3.1

For a complete graph $K_n^\sigma$ with $\sigma =n$, the first Zagreb spectrum is given by $$\begin{pmatrix} 0 & 2n(n+1) \\ n-1 & 1 \end{pmatrix}$$, where the first row entries are the eigenvalues and the corresponding second row entries are their multiplicities. The first Zagreb energy is $4(n^2-1)$.

### Proof

Since $\sigma =n$, the degree of each vertex in $K_{n}^{\sigma }$ is $n+1$. Thus, the first Zagreb matrix of $K_{n}^{\sigma }$ is $2(n+1)J_{n}$. Thus the first Zagreb spectrum of $K_{n}^{\sigma }$ consists of $2n(n+1)$ with multiplicity 1, and 0 with multiplicity $n-1$. Therefore, $\mathcal {E}_{Z_{1}}(K_{n}^{\sigma })=\sum _{i=1}^{n}|\zeta _{i}(K_{n}^{\sigma })-2(n+1)|=4(n^{2}-1)$. $\square$

The theorem below gives the first Zagreb spectrum of $K_{m,n}$ with $\sigma$ self-loops.

### Theorem 3.2

Let $K_{m,n}$ be the complete bipartite graph with partite sets *M* and *N* of size *m* and *n*, respectively. Suppose that $\sigma$ self-loops are added in total, with $1\le \sigma _{1}<m$ and $1\le \sigma _{2}<n$ self-loops in partite *M* and *N*, respectively. Then the spectrum of $Z_1(K^\sigma _{m,n})$ is $2n+4$, $2m+4$, and 0 with multiplicity at least $\sigma _{1}-1$, $\sigma _{2}-1$, and $m+n-\sigma -2$, respectively and the remaining four eigenvalues of $Z_1$ are the eigenvalues of *Q*, where *Q* is as given in Eq. (3.3).

### Proof

For a complete bipartite graph $K_{m,n}$ with $\sigma =\sigma _{1}+\sigma _2$ self-loops, with $1\le \sigma _{1}<m$ and $1\le \sigma _{2}<n$, we have $Z_1(K^\sigma _{m,n})=$

$$\begin{bmatrix} (2n+4)I_{\sigma _1 \times \sigma _1} & {\textbf {0}} & (m+n+4)J_{\sigma _1 \times \sigma _2} & (m+n+2)J_{ \sigma _1 \times (n-\sigma _2) }\\ {\textbf {0}} & {\textbf {0}} & (m+n+2)J_{(m-\sigma _1)\times \sigma _2} & (m+n)J_{(m-\sigma _1)\times (n-\sigma _2)}\\ (m+n+4)J_{\sigma _2 \times \sigma _1} & (m+n+2)J_{\sigma _2\times (m-\sigma _1)} & (2m+4)I_{\sigma _2 \times \sigma _2} & {\textbf {0}}\\ (m+n+2)J_{(n-\sigma _2)\times \sigma _1} & (m+n)J_{(n-\sigma _2)\times (m-\sigma _1)} & {\textbf {0}} & {\textbf {0}} \end{bmatrix}.$$

It is straightforward to see that $2n+4$, $2m+4$, and 0 are the eigenvalues of the matrix $Z_1$ with multiplicity at least $\sigma _{1}-1$, $\sigma _{2}-1$, and $m+n-\sigma -2$, respectively. Thus, we have obtained $m+n-4$ eigenvalues of $Z_1$. Now, note that the matrix $Z_1$ is equitable with quotient matrix $Q=$

3.3 $$\begin{aligned} \begin{bmatrix} 2n+4 & 0 & \sigma _2(m+n+4) & (n-\sigma _2)(m+n+2)\\ 0 & 0 & \sigma _2(m+n+2)& (n-\sigma _2)(m+n)\\ \sigma _1(m+n+4) & (m-\sigma _1) (m+n+2) & (2m+4) & 0\\ \sigma _1(m+n+2) & (m-\sigma _1)(m+n)& 0 & 0 \end{bmatrix}. \end{aligned}$$

Therefore, the remaining four eigenvalues of $Z_1$ are the eigenvalues of *Q*. $\square$

### Corollary 3.2.1

Let *G* be the complete bipartite graph $K_{m,n}$ with partite sets *M* and *N* of size *m* and *n*, respectively. Let $S=M$, then the first Zagreb energy of $G_S$ is given by

$$\begin{aligned} \mathcal {E}_{ Z_1}(G_S)=&\dfrac{4n^2m+6mn-2n^2-4n-4m}{m+n}\\ &+2\sqrt{m^3n+2m^2n^2+mn^3+4m^2n+4mn^2+4mn+n^2+4n+4}. \end{aligned}$$.

### Proof

From Theorem 3.2, the spectrum of $Z_1(K_{m,n}^m)$ is given by

$$\begin{pmatrix} 0 & 2n+4 & \zeta _1 & \zeta _2 \\ n-1 & m-1 & 1 & 1 \end{pmatrix}.$$

The first row contains the eigenvalues, and the second row lists their corresponding multiplicities. Here, $\zeta _1$ and $\zeta _2$ are given by $n+2\pm \sqrt{m^3n+2m^2n^2+mn^3+4m^2n+4mn^2+4mn+n^2+4n+4}$.

Therefore,

$$\begin{aligned}\mathcal {E}_{ Z_1}(K_{m,n}^m)&=(n-1)\Big ( \dfrac{m(2n+4)}{m+n}\Big )+(m-1)\Big |(2n+4)- \dfrac{m(2n+4)}{m+n}\Big |\\ &+\Big | \zeta _1- \dfrac{m(2n+4)}{m+n}\Big |+\Big | \zeta _2- \dfrac{m(2n+4)}{m+n}\Big |. \end{aligned}$$

On simplification, we get

$$\begin{aligned}\mathcal {E}_{ Z_1}(K_{m,n}^m)&=\dfrac{4n^2m+6mn-2n^2-4n-4m}{m+n}\\ &+2\sqrt{m^3n+2m^2n^2+mn^3+4m^2n+4mn^2+4mn+n^2+4n+4}. \end{aligned}$$

$$\square$$

## Bounds for the energy of $Z_1(G_S)$

In this section, we present some bounds for $\mathcal {E}_{ Z_1}(G_S)$. The forgotten index of a self-loop graph $G_{S}$ is denoted by $F(G_{S})$, and is given by $F(G_{S})=\sum _{v_{i} v_{j}\in E(G_{S})} {d^{\prime }}^{2}_{i}+{d^{\prime }}^{2}_{j}$. The second Zagreb index $M_{2}(G_{S})$ of $G_{S}$ is defined as $M_{2}(G_{S})=\sum _{v_{i} v_{j}\in E(G_{S})}d_{i}^{\prime }d_{j}^\prime$. The following lemmas follow form the elementary eigenvalue properties.

### Lemma 4.1

Let *G* be a graph on *n* vertices and let $S \subseteq V(G)$. Then

$$(i)\sum \limits _{i=1}^{n} \zeta _i=2\mathcal {D}_S$$

$$(ii)\sum \limits _{i=1}^{n}{ \zeta _i}^2=2F(G_S)+4M_2(G_S)+4M'_1(G_S)$$,

where $$\mathcal {D}_S=\sum \limits _{v_i \in S}d^{\prime }_i$$, and $$M_{1}^{\prime }(G_{S})=\sum _{v_{i}\in S}{d_{i}^{\prime }}^{2}$$.

### Proof

Since $trace(Z_{1}(G_{S}))=2\mathcal {D}_S$ and $trace(Z_{1}(G_{S})^2)=2F(G_S)+4M_2(G_S)+4M'_1(G_S)$, where $\mathcal {D}_S=\sum \limits _{v_i \in S}d^{\prime }_i$ and $M_{1}^{\prime }(G_{S})=\sum _{v_{i}\in S}{d_{i}^{\prime }}^{2}$, the proofs of (i) and (ii) follow from the facts that $\sum _{i=1}^{n}\zeta _{i}=trace(Z_{1}(G_S))$ and $\sum _{i=1}^{n}\zeta _{i}^{2}=trace(Z_{1}(G_S)^{2})$, respectively.

$$\square$$

### Lemma 4.2

Let *G* be a graph on n vertices and let $S \subseteq V(G)$. Let $\zeta ^*_i$ be the *i*-th largest real number in the sequence $\Bigl \{\Big |\zeta _i-\dfrac{2\mathcal {D_S}}{n}\Big |\Bigr \}$ for $i=1,2,\dots ,n$. Then

$$\begin{aligned} \sum \limits _{i=1}^{n}{\zeta _{i}^*}^2=2F(G_S)+4M_2(G_S)+4M'_1(G_S)- \dfrac{4\mathcal {D}_S^2}{n}. \end{aligned}$$

### Proof

The proof follows from the facts that $\sum _{i=1}^{n}{\zeta _{i}^{*}}^{2}=trace(Z_{1}(G_{S})-\dfrac{2D_{S}}{n}I)^{2}$ and

$$trace(Z_{1}(G_{S})-\dfrac{2D_{S}}{n}I)^{2}=2F(G_S)+4M_2(G_S)+4M'_1(G_S)- \dfrac{4\mathcal {D}_S^2}{n}$$.

$$\square$$

### Lemma 4.3

Let $G_{S}$ be a self-loop graph on *n* vertices. Then $Z_{1}(G_{S})$ is positive semi-definite if and only if the components of $G_{S}$ are either $K_{1}^{\sigma }$ with $\sigma =0$ or 1, or $K_{n_{1}}^{\sigma }$ with $\sigma =n_{1}>1$.

### Proof

Suppose $Z_{1}(G_{S})$ is positive semi-definite. Let $\Gamma _{S}$ be a connected component of $G_{S}$ of order $n_{1}$. Then $Z_{1}(\Gamma _{S})$ is positive semi-definite. If $\Gamma \cong K_{1}$, then there is nothing to prove. Otherwise, $\Gamma$ has at least one edge, say $v_{i}v_{j}\in E(\Gamma )$, then the second order principal submatrix of $Z_{1}(\Gamma _{S})$ corresponding to the vertices $v_{i}$ and $v_{j}$ is $$\begin{bmatrix}0& d_{i}+d_{j}\\ d_{i}+d_{j}& 0 \end{bmatrix}$$, $$\begin{bmatrix}2d_{i}+4& d_{i}+d_{j}+2\\ d_{i}+d_{j}+2& 0 \end{bmatrix}$$, or $$\begin{bmatrix}2d_{i}+4& d_{i}+d_{j}+4\\ d_{i}+d_{j}+4& 2d_{j}+4 \end{bmatrix}$$. It is straightforward to see that the first-two matrices are not positive semi-definite, and the last one is positive semidefinite if and only if $d_{i}=d_{j}$. Since $Z_{1}(\Gamma _{S})$ is positive semidefinite, by interlacing theorem, we must have both the vertices $v_{i}$ and $v_{j}$ in *S*, and $d_{i}=d_{j}$. Consequently, any two adjacent vertices in $\Gamma$ are in *S* and have the same degree. Note that $\Gamma$ is connected, therefore $\Gamma$ must be a $\gamma$-regular graph for some positive integer $\gamma$, and all its vertices are contained in *S*. Hence, $Z(\Gamma _{S})=2(\gamma +2) (A(\Gamma )+I_{n_{1}})$. We now claim that $\Gamma \cong K_{n_{1}}$. For if $\Gamma \ncong K_{n_{1}}$, then $\lambda _{n}(\Gamma )<-1$ and so $\zeta _{n_1}(\Gamma _{S})<0$, proving that $Z_{1}(\Gamma _{S})$ is not positive semidefinite, a contradiction. Thus, $\Gamma \cong K_{n_{1}}$. So, $\Gamma _{S}\cong K_{n_{1}}^{\sigma }$ with $\sigma =n_{1}$. Hence, a connected component of $G_{S}$ is either $K_{1}^{\sigma }$ with $\sigma =0$ or 1, or $K_{n_{1}}^{\sigma }$ with $\sigma =n_{1}>1$. Conversely, if $K_{1}^{\sigma }$ is a component of $G_{S}$, then 4 or 0 is a first Zagreb eigenvalue of $G_{S}$, and if $K_{n_{1}}^{\sigma }$ with $\sigma =n_{1}>1$ is a component of $G_{S}$, then $2n_{1}(n_{1}+1), \underset{n_{1}-1}{\underbrace{0,\ldots , 0}}$ are the first Zagreb eigenvalues of $G_{S}$. Thus, $Z_{1}(G_{S})$ is positive semidefinite if all the components of $G_{S}$ are either $K_{1}^{\sigma }$ with $\sigma =0$ or 1, or $K_{n_{1}}^{\sigma }$ with $\sigma =n_{1}>1$. $\square$

In the following theorem, we give an upper bound for $\mathcal {E}_{Z_{1}}(G_{S})$.

### Theorem 4.1

For a graph $G_{S}$ with n vertices and $\sigma$ self-loops, the first Zagreb energy $\mathcal {E}_{Z_{1}}(G_{S})$ is bounded above by

$$\begin{aligned} \zeta _1- \dfrac{2 \mathcal {D_S}}{n}+\sqrt{(n-1)\Big (2F(G_S)+4M_2(G_S)+4M'_1(G_S)- \zeta _1^2+ \dfrac{4(n-1) \mathcal {D_S}^2}{n^2}- \dfrac{4 \mathcal {D_S}}{n}(2 \mathcal {D_S}- \zeta _1)\Big )}. \end{aligned}$$

Equality holds if and only if the spectrum of $Z_{1}(G_{S})$ consists of $(t-s)a+\dfrac{2\mathcal {D}_{S}}{n}$, $\underset{s\,\, times}{\underbrace{a+\dfrac{2\mathcal {D}_{S}}{n},\ldots ,a+\dfrac{2\mathcal {D}_{S}}{n}}}$, and $\underset{t\,\, times}{\underbrace{-a+\dfrac{2\mathcal {D}_{S}}{n},\ldots ,-a+\dfrac{2\mathcal {D}_{S}}{n}}}$, where $s+t+1=n$, $t\ge s+1$, and $a\ge 0$.

### Proof

From Cauchy-Schwarz inequality, we have

4.1 $$\begin{aligned} \Big (\sum \limits _{i=2}^{n}\Big | \zeta _i- \dfrac{2 \mathcal {D_S}}{n}\Big |\Big )^2\le&\Big (\sum \limits _{i=2}^{n}1\Big )\Big (\sum \limits _{i=2}^{n}\Big | \zeta _i- \dfrac{2 \mathcal {D_S}}{n}\Big |^2\Big ). \end{aligned}$$

Employing Lemmas 4.1 and 4.2 in (4.1), we obtain

$$\begin{aligned} \Big (\mathcal {E}_{ Z_1}(G_S)- \Big | \zeta _1- \dfrac{2 \mathcal {D_S}}{n}\Big |\Big )^2\le&(n-1) \Big (\sum \limits _{i=2}^{n} \zeta _i^2+ \dfrac{4(n-1) \mathcal {D_S}^2}{n^2}-\dfrac{4 \mathcal {D_S}}{n} \sum \limits _{i=2}^{n} \zeta _i\Big ) \\ =&(n-1)\Big (2F(G_S)+4M_2(G_S)+4M'_1(G_S)- \zeta _1^2\\ &+ \dfrac{4(n-1) \mathcal {D_S}^2}{n^2}- \dfrac{4 \mathcal {D_S}}{n}(2 \mathcal {D_S}- \zeta _1)\Big ). \end{aligned}$$

Therefore,

$$\begin{aligned} \mathcal {E}_{ Z_1}(G_S)\le \zeta _1- \dfrac{2 \mathcal {D_S}}{n}+\sqrt{(n-1)\Big (2F(G_S)+4M_2(G_S)+4M'_1(G_S)- \zeta _1^2+ \dfrac{4(n-1) \mathcal {D_S}^2}{n^2}- \dfrac{4 \mathcal {D_S}}{n}(2 \mathcal {D_S}- \zeta _1)\Big )}. \end{aligned}$$

Note that the equality holds if and only if $|\zeta _{i}-\dfrac{2\mathcal {D}_{S}}{n}|=a$ for $i=2,3,\ldots n$, and $a\ge 0$. Thus, $a+\dfrac{2\mathcal {D}_{S}}{n}$ and $-a+\dfrac{2\mathcal {D}_{S}}{n}$ are the eigenvalues of $Z_{1}(G_{S})$, with multiplicity *s* and *t*, respectively. Since $\sum _{i=1}^{n}\left( \zeta _{i}-\dfrac{2\mathcal {D}_{S}}{n}\right) =0$ and $\zeta _{1}\ge \dfrac{2\mathcal {D}_{S}}{n}$, we must also have $t\ge s\ge 0$, and $\zeta _{1}=(t-s)a+\dfrac{2\mathcal {D}_{S}}{n}$. This completes the proof of the theorem. $\square$

### Remark 4.2

The upper bound in Theorem 4.1 is attained for the graphs $\overline{K_{n}}^{\sigma }$ with $\sigma =0, 1,$ and *n*; $K_{n}^{\sigma }$ with $\sigma =n$; and $\dfrac{n}{2}K_{2}^{\sigma _{1}}$ with $\sigma _{1}=0,1,2$.

### Theorem 4.3

For a graph *G* of order $n\ge 3$,

$$\begin{aligned} \mathcal {E}_{ Z_1}(G_S) \le \sqrt{2n(F(G_S)+2M_2(G_S)+2M'_1(G_S))- \dfrac{n}{2}(\zeta _1^*-\zeta _n^*)^2}. \end{aligned}$$

### Proof

Employing Lagrange’s identity to the vectors $(\zeta _1^*,\zeta _2^*,\dots ,\zeta _n^*)$ and $(\underbrace{1,1,\dots , 1}_{n})$, we get

4.2 $$\begin{aligned} n\sum \limits _{i=1}^{n}{\zeta _i^*}^2-\big (\sum \limits _{i=1}^{n}\zeta _i^*\big )^2&=\sum _{1 \le i< j \le n} (\zeta ^*_i- \zeta ^*_j )^2\nonumber \\&= \sum \limits _{i=2}^{n-1}\Big [(\zeta _1^*-\zeta _i^*)^2+(\zeta _i^*-\zeta _n^*)^2\Big ]+(\zeta _1^*-\zeta _n^*)^2\nonumber \\&+\sum _{2\le i<j\le n-1}(\zeta _i^*-\zeta _j^*)^2. \end{aligned}$$

For $i\ne 1,n$, we have

$$\begin{aligned}((\zeta _{1}^{*}-\zeta _{i}^{*})+(\zeta _{i}^{*}-\zeta _{n}^{*}))^{2}&=(\zeta _{1}^{*}-\zeta _{i}^{*})^2+(\zeta _{i}^{*}-\zeta _{n}^{*})^2+2(\zeta _{1}^{*}-\zeta _{i}^{*})(\zeta _{i}^{*}-\zeta _{n}^{*})\\&\le (\zeta _{1}^{*}-\zeta _{i}^{*})^2+(\zeta _{i}^{*}-\zeta _{n}^{*})^2+\left( \dfrac{\zeta _{1}^{*}-\zeta _{i}^{*}}{\zeta _{i}^{*}-\zeta _{n}^{*}}+\dfrac{\zeta _{i}^{*}-\zeta _{n}^{*}}{\zeta _{1}^{*}-\zeta _{i}^{*}}\right) (\zeta _{1}^{*}-\zeta _{i}^{*})(\zeta _{i}^{*}-\zeta _{n}^{*})\\&=2\left[ (\zeta _{1}^{*}-\zeta _{i}^{*})^2+(\zeta _{i}^{*}-\zeta _{n}^{*})^2\right] . \end{aligned}$$

Thus,

4.3 $$\begin{aligned} \dfrac{(\zeta _{1}^{*}-\zeta _{n}^{*})^2}{2}\le (\zeta _{1}^{*}-\zeta _{i}^{*})^2+(\zeta _{i}^{*}-\zeta _{n}^{*})^2.\end{aligned}$$

Employing Eqs. (4.3) in (4.2), and subsequently applying Lemma 4.2, we obtain

$$\begin{aligned} n\Theta -(\mathcal {E}_{ Z_1}(G_S))^2&\ge \dfrac{n(\zeta _1^*-\zeta _n^*)^2}{2}, \quad where\,\, \Theta = 2\Big (F(G_S)+2M_2(G_S)+2M'_1(G_S)- \dfrac{2\mathcal {D}_S^2}{n}\Big ). \end{aligned}$$

Hence,

4.4 $$\begin{aligned} \mathcal {E}_{ Z_1}(G_S)&\le \sqrt{n\Theta -\dfrac{n(\zeta _1^*-\zeta _n^*)^2}{2}}. \end{aligned}$$

Equality holds if and only if $\sum _{2\le i<j\le n-1}(\zeta _i^*-\zeta _j^*)^2=0$, and for $i\ne 1,n$, $\zeta _1^*-\zeta _i^*$=$\zeta _i^*-\zeta _n^*$. Therefore, the equality in (4.4) holds, if and only if $\zeta _{2}^{*}=\zeta _{3}^{*}=\cdots =\zeta _{n-1}^{*}=\dfrac{\zeta _{1}^{*}+\zeta _{n}^{*}}{2}$. $\square$

The following theorems give lower bounds for $\mathcal {E}_{Z_{1}}(G)$.

### Theorem 4.4

For a graph *G* of order $n\ge 3$,

$$\begin{aligned} \mathcal {E}_{ Z_1}(G_S)\ge \sqrt{2\Theta +n(n-1)\Big |det(Z_1(G_S)-\dfrac{2\mathcal {D_S}}{n}I)\Big |^{\dfrac{2}{n}}}. \end{aligned}$$

Equality holds if and only if $G_{S}\cong \overline{K_{n}}^{\sigma }$ with $\sigma =0,n,\dfrac{n}{2}$, or $G_{S}\cong \dfrac{n}{2}K_{2}^{\sigma _{1}}$, where $0\le \sigma _{1}\le 2$.

### Proof

Applying AM-GM inequality for the real numbers $\zeta _i^*\,\zeta _j^*$, where $1\le i<j\le n$, we get

$$\begin{aligned} \dfrac{2}{n(n-1)}\sum \zeta _i^*\zeta _j^*&\ge \prod (\zeta _i^*\zeta _j^*)^{\dfrac{2}{n(n-1)}}. \end{aligned}$$

Upon simplification,

$$\begin{aligned} \dfrac{1}{n(n-1)}\sum \zeta _i^*(\mathcal {E}_{ Z_1}(G_S)-\zeta _i^*) \ge&\prod {\zeta _i^{*}}^{\dfrac{2}{n}}. \end{aligned}$$

That is,

$$\begin{aligned} \dfrac{1}{n(n-1)}\big [\mathcal {E}^2_{ Z_1}(G_S)-\Theta \big ]&\ge \Big |det(Z_1(G_S)-\dfrac{2\mathcal {D_S}}{n}I)\Big |^{\dfrac{2}{n}},\, \text {where} \end{aligned}$$

$$\Theta =2\Big (F(G_S)+2M_2(G_S)+2M'_1(G_S)- \dfrac{2\mathcal {D}_S^2}{n}\Big ).$$

Therefore,

$$\begin{aligned} \mathcal {E}_{ Z_1}(G_S)\ge \sqrt{\Theta +n(n-1)\Big |det(Z_1(G_S)-\dfrac{2\mathcal {D_S}}{n}I)\Big |^{\dfrac{2}{n}}}. \end{aligned}$$

Suppose the equality holds. Then $|\zeta _{i}^{*}\zeta _{j}^{*}|=|\zeta _{k}^{*}\zeta _{l}^{*}|$ for all $1\le i<j\le n$ and $1\le k<l\le n$. Thus, $\zeta ^{*}_{1}=\zeta ^{*}_{2}=\cdots =\zeta _{n}^{*}=a (> 0)$, or $\zeta ^{*}_{2}=\cdots =\zeta _{n}^{*}=0$.

Case 1: $\zeta ^{*}_{1}=\zeta ^{*}_{2}=\cdots =\zeta _{n}^{*}=a (> 0)$. Then the spectrum of $Z_{1}(G_{S})$ consists of $a+\dfrac{2\mathcal {D}_{S}}{n}$, and $-a+\dfrac{2\mathcal {D}_{S}}{n}$ each with multiplicity $\dfrac{n}{2}$ ( since $\sum _{i=1}^{n}\left( \zeta _{i}-\dfrac{2\mathcal {D}_{S}}{n}\right) =0$, and so the number of *a*’s in the sum must be equal to the number of $-a$’s ).

Subcase 1: $-a+\dfrac{2\mathcal {D}_{S}}{n}\ge 0$. Then $Z_{1}(G_{S})$ is positive semi-definite, and so by Lemma 4.3, the components of $G_{S}$ are either $K_{1}^{\sigma _{1}}$ with $\sigma _{1}=0,1$; or $K_{n_1}^{\sigma _{2}}$ with $\sigma _{2}=n_{1}>1$. Since $Z_{1}(G_{S})$ has exactly two distinct eigenvalues, namely $a+\dfrac{2\mathcal {D}_{S}}{n}$ and $-a+\dfrac{2\mathcal {D}_{S}}{n}$ each with multiplicity $\dfrac{n}{2}$, it is easy to see that $G_{S}\cong \overline{K_{n}}^{\sigma }$, with $\sigma =\dfrac{n}{2}$; or $G_{S}\cong \dfrac{n}{2}K_{2}^{\sigma _{1}}$, where $\sigma _{1}=2$.

Subcase 2: $-a+\dfrac{2\mathcal {D}_{S}}{n}< 0$. By Perron Frobenius theorem, the largest eigenvalue of a non-negative irreducible matrix is simple. Since $a+\dfrac{2\mathcal {D}_{S}}{n}$ is the largest eigenvalue of $G_{S}$ with multiplicity $\dfrac{n}{2}$, it follows that *G* has exactly $\dfrac{n}{2}$ components, each of order 2. Thus, $G_{S}\cong \dfrac{n}{2}K_{2}^{\sigma _{1}}$, where $0\le \sigma _{1}\le 1$.

Case 2: $\zeta ^{*}_{2}=\cdots \zeta _{n}^{*}=0$. Since $\sum _{i=1}^{n}\left( \zeta _{i}-\dfrac{2\mathcal {D}_{S}}{n}\right) =0$, we must have $\zeta ^{*}_{1}=0$. Thus, $\zeta _{i}=\dfrac{2\mathcal {D}_{S}}{n}$ for all $1\le i\le n$. So, $Z_{1}(G_{S})$ is positive semi-definite. Bearing Lemma 4.3 in mind, and since all the $\zeta _{i}$’s are equal, we find that $G_{S}\cong \overline{K_{n}}^{\sigma }$ with $\sigma =0$, or $\sigma =n$.

Converse part is direct. $\square$

### Theorem 4.5

For a graph *G* of order $n\ge 3$,

$$\begin{aligned} \mathcal {E}_{ Z_1}(G_S)\ge 2\sqrt{F(G_S)+2M_2(G_S)+2M'_1(G_S)- \dfrac{2\mathcal {D}_S^2}{n}}. \end{aligned}$$

Equality holds if and only if $G\cong K_{n}^{\sigma }$ with $\sigma =0$, or $\sigma =n$.

### Proof

Let $\zeta _i'=\zeta _i-\dfrac{2\mathcal {D_S}}{n}$ for $i=1,2,\dots ,n$. Then $\sum _{i=1}^{n}\zeta _i'=0,$ and so $\big (\sum \limits _{i=1}^{n} \zeta _i'\big )^2=0.$ This implies, $\sum \limits _{i=1}^{n}\zeta _i'^2+2\sum \limits _{i<j} \zeta _i'\zeta _j'=0$. Hence

4.5 $$\begin{aligned} \sum \limits _{i=1}^{n} \zeta _i'^2=-2\sum \limits _{i<j} \zeta _i'\zeta _j'.\end{aligned}$$

Now,

$$\begin{aligned} \mathcal {E}_{ Z_1}(G_S)^2&=\Big (\sum \limits _{i=1}^{n} | \zeta _i'|\Big )^2 =\sum \limits _{i=1}^{n} \zeta _i'^2+2\sum \limits _{i<j}| \zeta _i'||\zeta _j'|\\&\ge \sum \limits _{i=1}^{n} \zeta _i'^2+2\Big |\sum \limits _{i<j} \zeta _i'\zeta _j'\Big |\quad \big [\text {by\, triangular\, inequality}\big ]\\&=\sum \limits _{i=1}^{n} \zeta _i'^2+\sum \limits _{i=1}^{n} \zeta _i'^2 \quad \big [\text {see\, equation\, (4.5)}\big ]\\&=2\sum \limits _{i=1}^{n} \zeta _i'^2. \end{aligned}$$

Thus, by Lemma 4.2, we have

4.6 $$\begin{aligned} \mathcal {E}_{ Z_1}(G_S)\ge 2\sqrt{\Big (F(G_S)+2M_2(G_S)+2M'_1(G_S)- \dfrac{2\mathcal {D}_S^2}{n}\Big )}. \end{aligned}$$

Suppose the equality in the Eq. (4.6) holds. Then $\sum _{i<j}|\zeta _{i}^{\prime }||\zeta _{j}^{\prime }|=\big |\sum _{i<j}\zeta _{i}^{\prime }\zeta _{j}^{\prime }\big |$, which is true if and only if $\zeta ^{\prime }_{i}\zeta ^{\prime }_{j}\ge 0$ for all $1\le i<j\le n$. By Rayleigh quotient inequality, we find that

4.7 $$\begin{aligned} \zeta _{1}\ge 2\left( \dfrac{\mathcal {D}_{S}}{n}+\sum _{\underset{i\sim j}{i\ne j}}\left( d_{i}^{\prime }+d_{j}^{\prime }\right) \right) . \end{aligned}$$

Therefore, $\zeta _{1}^{\prime }\ge 0$. Now, if $\zeta ^{\prime }_{1}>0$. Then $\zeta ^{\prime }_{i}<0$ for some $1<i\le n$ because $\sum _{i=1}^{n}\zeta _{i}^{\prime }=0$. So, $\zeta _{1}^{\prime }\zeta _{i}^{\prime }<0$, and thus the inequality in Eq. (4.6) is strict. Hence, $\zeta ^{\prime }_{1}=0$. Now, by Eq. (4.7), we get $\sum _{\underset{i\sim j}{i\ne j}}\left( d_{i}^{\prime }+d_{j}^{\prime }\right) =0$. This implies, *G* is a totally disconnected graph, i.e., $G\cong \overline{K_{n}}$. Further, if $G\cong \overline{K_{n}}$, and $1<\sigma <n$, then $\zeta _{1}^{\prime }=\zeta _{2}^{\prime }=\cdots =\zeta _{\sigma }^{\prime }=4\left( 1-\dfrac{\sigma }{n}\right)$, and $\zeta _{\sigma +1}^{\prime }=\zeta _{\sigma +2}^{\prime }=\cdots =\zeta _{n}^{\prime }=-\dfrac{4\sigma }{n}.$ Thus, $\zeta _{1}^{\prime }\zeta _{n}^{\prime }<0$. Proving that, $G_{S}\cong K_{n}^{\sigma }$ with $\sigma =0$, or $\sigma =n$. $\square$

## Future work

The study of the first Zagreb energy of self-looped graphs can be extended in several promising directions. One of the interesting problems is to determine the maximum first Zagreb energy among graphs $G_{S}$, where the underlying graph *G* is fixed and *S* is a vertex subset of fixed cardinality. Characterizing extremal subsets *S* that maximize the Zagreb energy would provide further insight into the influence of self-loops on spectral properties. Further investigation can also focus on exploring the relationship between the first Zagreb index of $G_{S}$ and the first Zagreb energy of *G*. The concept of the first Zagreb energy for self-loop graphs can be extended to other classes of degree-based topological matrices, to understand how self-loops influence spectral properties and graph invariants. In addition, the chemical applicability of the first Zagreb energy of self-looped graphs can be broadened by conducting quantitative structure–property relationship (QSPR) analysis on a wider class of drug molecules. Machine learning methods may be adopted to improve predictive accuracy and to further validate the effectiveness of the descriptor. Such studies may help assess its effectiveness as a molecular descriptor and strengthen its relevance in mathematical chemistry.

## Conclusion

In this study, the first Zagreb matrix and the associated energy for graphs with self-loops were introduced and analyzed. The correlation between the first Zagreb energy $\mathcal {E}_{ Z_1}(G_S)$ and various physicochemical properties of kidney infection drugs was examined using multiple regression models, including linear, quadratic, cubic, and logarithmic regressions. The analysis revealed that $\mathcal {E}_{ Z_1}(G_S)$ serves as an effective structural descriptor, exhibiting strong correlations–particularly with molar refractivity ($MR$), polarizability ($P$), and molar volume ($MV$). These results indicate that the inclusion of self-loops enhances the predictive capability of the $\mathcal {E}_{ Z_1}(G_S)$ compared to the $\mathcal {E}_{ Z_1}(G)$. Additionally, the spectrum and energy of complete and complete bipartite graphs with self-loops were derived, along with several upper and lower bounds for $\mathcal {E}_{ Z_1}(G_S)$.

## Data Availability

The datasets analysed during the current study are available in the ChemSpider repository, https://share.google/vZ1eZe5e60JRlhIpW. The regression analysis was carried out through Microsoft Excel, and the necessary computations were performed using MATLAB R2024a.
